# Retrospective data analyses of social and environmental determinants of malaria control for elimination prospects in Eritrea

**DOI:** 10.1186/s13071-020-3974-x

**Published:** 2020-03-12

**Authors:** Selam Mihreteab, Jailos Lubinda, Bingxin Zhao, Alfonso J. Rodriguez-Morales, Ajlina Karamehic-Muratovic, Aman Goitom, Muhammad Yousaf Shad, Ubydul Haque

**Affiliations:** 1 National Malaria Control Programme, Ministry of Health, Asmara, Eritrea; 2 School of Geography and Environmental Sciences, Ulster University, Coleraine, UK; 3 Department of Biostatistics, University of North Carolina at Chapel Hill, Chapel Hill, NC USA; 4 Public Health and Infection Research Group, Faculty of Health Sciences, Universidad Tecnológica de Pereira, Pereira, Risaralda Colombia; 5 Medical School, Faculty of Health Sciences, UniFranz, Cochabamba, Bolivia; 6 Grupo de Investigación Biomedicina, Faculty of Medicine, Fundación Universitaria Autónoma de las Américas, Pereira, Risaralda, Colombia; 7 Department of Sociology and Anthropology, St Louis University, St. Louis, MO USA; 8 Department of Statistics, Quaid-i-Azam University, Islamabad, Pakistan; 9 Department of Geography, University of Florida, Gainesville, FL USA; 10 Emerging Pathogens Institute, University of Florida, Gainesville, FL USA; 11 Department of Biostatistics and Epidemiology, University of North Texas Health Science Center, Fort Worth, TX USA

**Keywords:** Malaria, Vector-borne diseases, Public health, Control, Elimination, Eritrea

## Abstract

**Background:**

The present study focuses on both long- and short-term malaria transmission in Eritrea and investigates the risk factors. Annual aggregates of information on malaria cases, deaths, diagnostics and control interventions from 2001 to 2008 and monthly reported data from 2009 to 2017 were obtained from the National Malaria Control Programme. We used a generalized linear regression model to examine the associations among total malaria cases, death, insecticide-treated net coverage, indoor residual spraying and climatic parameters.

**Results:**

Reduction in malaria mortality is demonstrated by the milestone margins of over 97% by the end of 2017. Malaria incidence likewise declined during the period (from 33 to 5 per 1000 population), representing a reduction of about 86% (*R*^2^ = 0.3) slightly less than the decline in mortality. The distribution of insecticide treated nets generally declined between 2001 and 2014 (*R*^2^ = 0.16) before increasing from 2015 to 2017, while the number of people protected by indoor residual spraying slightly increased (*R*^2^ = 0.27). Higher rainfall was significantly associated with an increased number of malaria cases. The covariates rainfall and temperature are a better pair than IRS and LLIN to predict incidences. On the other hand, IRS and LLIN is a more significant pair to predict mortality cases.

**Conclusions:**

While Eritrea has made significant progress towards malaria elimination, this progress should be maintained and further improved. Distribution, coverage and utilization of malaria control and elimination tools should be optimized and sustained to safeguard the gains made. Additionally, consistent annual performance evaluation of malaria indicators would ensure a continuous learning process from gains/threats of epidemics and resurgence in regions already earmarked for elimination.

## Background

Following a nationwide malaria epidemic in 1998, Eritrea has made remarkable progress on basic health indices [1], most notably in reducing malaria incidence (80%) and mortality (90%) by 2015 compared with the 1998 baseline [2]. This progress placed Eritrea in the spotlight as one of Africa’s model countries in malaria control. In 2016, the country was one of the eight recipients of the African Leaders Malaria Alliance Award (ALMA) for excellence in achieving the Millennium Development Goals (MDGs) target for malaria [3].

Through the Ministry of Health, Eritrea established a Health Management Information System (HMIS) [4] and joined other African countries in calling for a malaria-free continent by 2030. The World Health Organization (WHO) Global Technical Strategy for Malaria 2016–2030 was launched towards this effort [5]. Eritrea’s target for malaria incidence and mortality reduction is 40% and 75% by 2020 and 2025, respectively, with the long-term goal to ultimately eliminate and prevent re-establishment of malaria by 2030 [6].

Incidence of malaria tends to peak in October in most zobas, while the months March-April mark the main transmission season in the coastal areas. The most common malaria parasite is *Plasmodium falciparum* which accounts for roughly 67% of all malaria cases [7]. *Plasmodium vivax* accounts for nearly 30% and few proportions of mixed infections [7]. *Anopheles arabiensis* is the main vector of malaria in the country followed by *An. d’thali*, *An. cinereus*, *An. rhodesiensis*, *An. squamosus* and *An. rupicolus* [8].

Established in 1995, the National Malaria Control Programme (NMCP) of Eritrea drafted a strategy of control program by 1997 (Additional file 1: Table S1). In 2000, the NMCP also launched the second national malaria control programme. In 2001, the NMCP conducted the national malaria prevalence survey and planned for malaria interventions (Additional file 1: Table S1). In 2007, the NMCP introduced RDT at the community level and on 2016 HRP2 based RDTs replaced with Pf-pLDH/PanpLDH RDTs. A combination of artesunate + amodiaquine was introduced as a first-line treatment for uncomplicated malaria in 2007 and Artesunate injection was introduced in 2016 for treatment of severe malaria cases. Over the years, the NMCP drafted and revised many policy guidelines to control malaria in Eritrea (Additional file 1: Table S1).

This study is an early assessment of Eritrea’s National Malaria Control Programme. It reflects Eritrea’s efforts in achieving the needed incremental progress towards 2020, 2025 and 2030 goals. We reviewed the progress made from 2001 to 2017, analyzed risk factors using 2009–2017 monthly reported data, discussed the major strategic efforts and implications of interventions implemented and investigated the role of climatic parameters in the spread of malaria cases in Eritrea. This study also extracted patterns of yearly progress to capture short-term oscillations that eventually tie in with the prospecting planned milestones towards elimination.

## Methods

### Study area

**Fig. 1:**
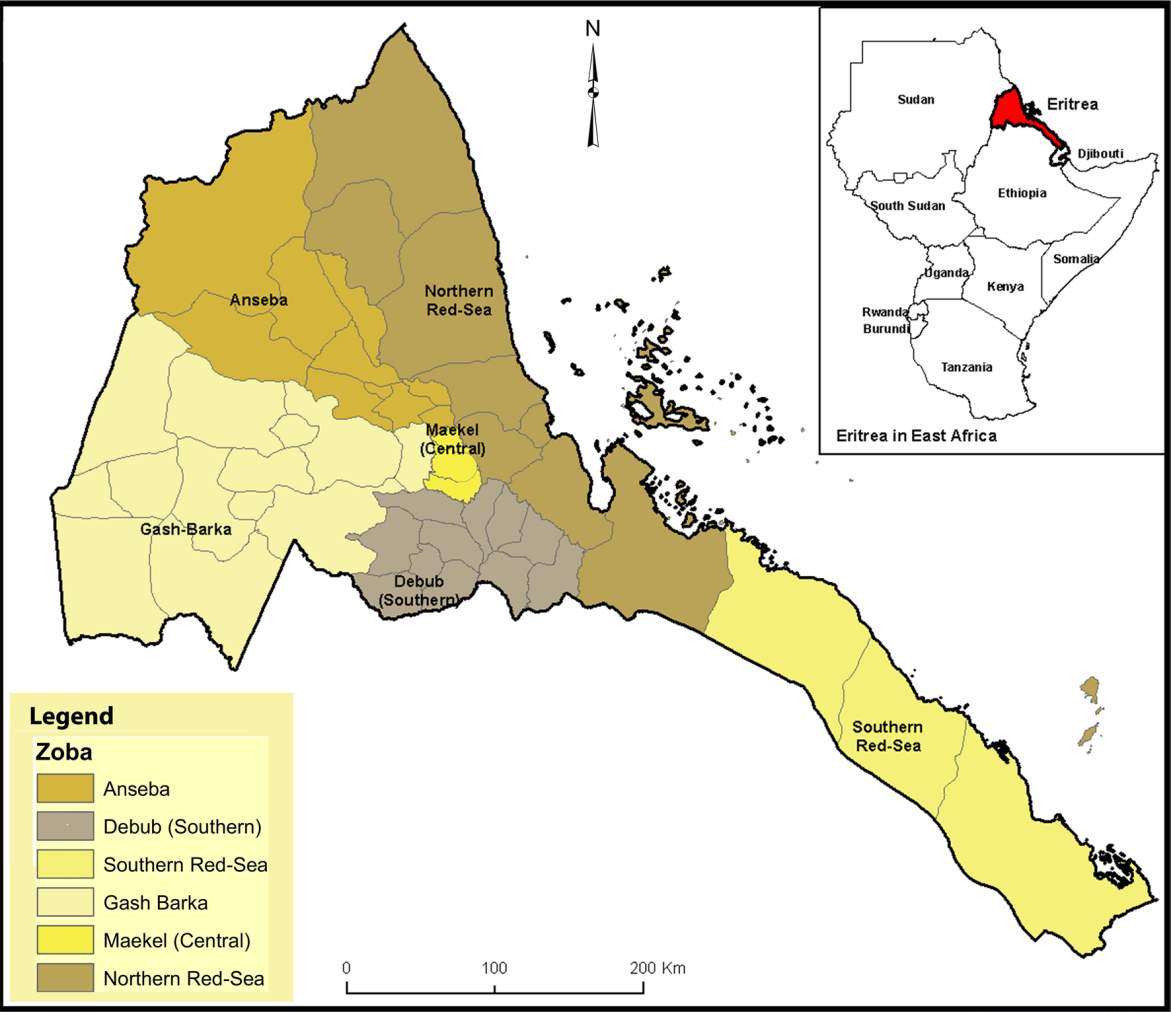
Location of Eritrea in Africa

Eritrea is located to the south-west of the Red Sea, bordered by Ethiopia to the south, Sudan to the north and west, and the Republic of Djibouti to the south-west (Fig. 1) [9]. Nearly 5 million people live in 124,000 square kilometers [10, 11]. The country is administratively divided into six geographical regions called zobas (Fig. 1) [1, 11, 12]. Malaria is highly seasonal and is prone to epidemics in the western lowlands. Transmission is perennial along rivers, valleys, dams and irrigation projects. The coastal plains have similar malaria situations to the western lowlands, but with notably less precipitation. The highlands are generally free from malaria but are highly prone to malaria epidemics because of the low immunity of these populations. Approximately 70% of the estimated population (nearly 3.5 million) resides in malaria-endemic areas.

### Data sources

The Ministry of National Development provided all population and projections data from 2001 to 2017 at a growth rate of 2.8%. We obtained malaria epidemiology data from the HMIS, Eritrea. Diagnosis (Additional file 2: Text S1), long-lasting insecticidal net (LLINs) and indoor residual spraying (IRS) data were obtained from the National Malaria Control Programme (NMCP). Health workers at their respective facilities fill different types of registers on a routine daily basis and complete a monthly HMIS summary form. Monthly HMIS summary forms are then sent to a sub-zoba, to which the facility belongs. The HMIS office at sub-zoba retains a copy and sends all monthly HMIS summary forms (i.e. for all facilities administered under it) to the HMIS office at zoba. Referral hospitals electronically send monthly data. Thus, the National HMIS collates monthly data from zobas and referral hospitals electronically. The ministry routinely collects malaria data from out-patient and in-patient facilities including health stations, health centers, district hospitals and referral hospitals throughout the country, with over 90% completeness and timeliness in reporting [13]. We downloaded the shapefiles from DIVA-GIS (freely available, http://www.diva-gis.org) and analyzed the spatial and temporal data comprised of sub-zoba level aggregations of malaria incidence, mortality, diagnostics and intervention coverage for LLINs from 2001 to 2017. We also used detailed sub-national (district/sub-zoba) incidence and mortality outpatient and inpatient data, as well as sub-zoba level of IRS and LLIN coverage in the period 2009–2017.

### Environmental data

Maximum and minimum daily temperature data (surface air temperature at 2 m height) were obtained for each location (sub-zoba) from the Climate Forecast System Reanalysis (CFSR) dataset of the National Centers for Environmental Prediction (NCEP) [14]. The CFSR is a global high-resolution (~ 33 km) hourly product available from 1979 to 2011, extended to the present as CFSR version 2 at a resolution of ~ 22 km [14].

Daily rainfall data were obtained for each location (sub-zoba) from the Climate Hazards Group Infrared Rainfall with Stations (CHIRPS) dataset developed by the United States Geological Survey (USGS) in collaboration with the Department of Geography, University of California, Santa Barbara [15]. The CHIRPS dataset is a high-resolution (~5 km) quasi-global (50°S to 50°N) rainfall climatology constructed from a combination of satellite imagery and *in-situ* data.

### Malaria morbidity, mortality, test positivity rate and diagnostics

We used population estimates as denominators to calculate malaria incidence, mortality, admission and test positivity rates. Malaria incidence and mortality rates were calculated per 1000 population. Test positivity rate (TPR) is the proportion of laboratory-confirmed malaria tests per 100 suspected cases examined. The diagnostic tools used to confirm malaria cases during the reported period included microscopy and rapid diagnosis tests (RDTs). In lower-level health facilities (namely health stations), clinical diagnosis was practiced by health providers lacking diagnostic tools over a decade ago [13], i.e. before 2007. At present, suspected malaria cases are rarely treated on clinical suspicion but only after confirmation with a parasitological diagnosis. In certain circumstances, suspected malaria patients may be treated with anti-malarial drugs in the absence of parasitological diagnosis or a negative result but when the clinician has a strong suspicion that the fever is due to malaria. Even if such a practice is exercised to save the life of the patient, parasitological testing must be repeated every 6–12 h to confirm and report the case as malaria or not.

LLINs coverage was computed using data aggregated from all distribution channels including mass campaigns, antenatal clinics, and other partners. A rate of two household residents per net calculated as

$$ {\text{\%  people covered by LLINs }} = \frac{{{\text{No}}.\,{\text{of LLINs distributed in the past}}\, 3\,{\text{years }} \times 2}}{\text{Population at risk}} $$

was used to calculate coverage as per Kilian et al. [16], Haque et al. [17] and Kamuliwo et al. [18]. Calculation of administrative coverage of LLINs was guided by the WHO Pesticide Evaluation Scheme testing process, which averages the net lifespan (decay of efficacy) at 3 years if the nets retain full efficacy given their minimum standard of insecticidal activity for this period. Decline in bed net coverage due to wear and tear, and loss is estimated at 8% during year 1, 20% in year 2 and 50% in year 3. One net is also distributed to 1.8 persons to compensate for odd household sizes (e.g. 2 nets, instead of 1.5 nets, are given to a household of 3 persons). The true decay and net loss may vary from place to place [19] and depend on access levels [16]. The percentage ratio of national level coverage and people protected by indoor residual spraying (IRS) was calculated as:

$$ \frac{{{\text{No}}.\;{\text{of structures }}\left[ {\text{actually}} \right]\,   {\text{sprayed }}}}{{{\text{No.}}  \;{\text{of structures planned }}\left[ {\text{targeted}} \right]}} \times 100 $$

and

$$ \frac{{{\text{No.}}\;{\text{of people }}\left[ {\text{actually}} \right]   {\text{protected }}}} {{{\text{No.}}\;{\text{of people }}\left[ {\text{targeted}} \right]}} \times 100 $$

from 2009 to 2017 [2].

### Spatial analysis

A spatial distribution of yearly malaria incidence map was created at sub-zoba level using ArcGIS v.10.2 software.

### Statistical analysis

We used a generalized linear (Poisson regression) model (GLM) to analyze data. This methodology used in many discrete cases predicted studies along with Bayesian STCAR and other GLM techniques. The response variable *Y* (number of deaths) was distributed as a Poisson random variable, the E(Y/ *x*) or log[E(Y/ *x*)] followed a linear model of covariates.

Now, *x* = (x_1_, x_2_, x_3_, …, x_k_) set of k independent predictors defined on a k-dimension real number space i.e. *x* ∈ R^k^. The log-link model discussed is written as1where  is the rate of occurrence of event (number of deaths etc.) and T is exposure time.

2 $$ \log \left( \lambda \right) = \alpha + \beta x $$

3

4 $$ {\text{E }}\left( {\text{response}} \right) \, = {\text{ Te}}^{\alpha + \beta x} $$

5 $$ {\text{E }}\left( {\text{response}} \right) \, = {\text{e}}^{{\log ({\text{T}}) + \alpha + \beta x}} $$

When X_i_ increases by 1 unit, log () will increase by β_i_. Thus  is multiplied by e^βi^ and e^β^ is known as incidence rate. Estimates of *α* and *β* were obtained by using (*Y*_*i*_, *x*_i_) available set of observations with the help of maximum likelihood estimator technique.

The probability mass function for the random variable *y* (count of deaths) following a Poisson distribution is given by

6 $$ { \Pr }({\text{Y}} = {\text{ y}}|x) \, = \frac{{e^{ - \lambda } \lambda^{y} }}{y!} $$

where7

A dataset of *n* vectors i.e. (*Y*_*i*_, *x*_i_) for i =1, 2, 3, …, n provided us estimates of *α* and *β* which makes the joint probability P (*y*_*1*_*, y*_*2*_*,…, y*_*n*_ / *x*_*i*_) as large as possible.

It was assumed malaria morbidity and mortality varies by space and time and might be associated with other covariates such as rainfall, temperature, IRS and LLIN coverage. The study variable is a discrete variable (number of cases, deaths). These are aggregated data at sub-zoba level. In the given circumstances, Poisson regression is more suitable.

8 $$ {\text{Log }}\left[ {{\text{E}}\left( {{\text{Y}}/x} \right)} \right] \, = \, \log \left( {\text{population}} \right) \, + \, \left( {\alpha + \beta x} \right) $$

This equation can be reduced to: log [E(Y/*x*)/ population] = *α* + *βx*

### Buishand change point detection test

Buishand U-test and Buishand range test for quantifying magnitude of change have been used in environmental change analysis from time series datasets [20]. While detecting variable change-point, we set the null hypothesis of zero shift in the data. This method considers the difference of maximum and minimum change in frequency-scaled over standard deviation as a test statistic. It marks the change as significant whenever it finds the test statistic significant which is the ratio of [Max(res)-Min(res)]/SD. Changing the set of sample data may produce a different result as even for a very small difference i.e. [Max(res)-Min(res)] may turn as significant if SD is very small. We tested the above null hypothesis and marked as change point when test statistic is significant. We used interrupted time series (ITS) complimented by the Buishand U-test and Buishand range test for change-point detection on national level data. We detected corroborated results on all probable change point at times using the *trend* package in R [21]. However, three variables (the number of people protected by IRS, number of LLINs distributed and TPR) were not significant at 95% CI in both tests. The results of the two change detection tests are provided in Additional file 3: Table S2. The ITS used a segmented regression model with formula:

$$ Y_{t} = \beta_{0} + \beta_{1} T + \beta_{2} X_{t} + \beta_{3} TX_{t} $$

where *β*_0_ is the baseline level at *T* = 0 and Xt = 0, *β*_1_ is the change-point of interruption in the outcome linked with a unit time increase after the pre-intervention trend. Meanwhile, *β*_2_ is the (step) level change (new level vertically below *β*_1_) subsequent to the intervention and *β*_3_ represents the slope change after the intervention viewed through the interaction of time and intervention (*TX*_*t*_).

## Results

### Changing decline in malaria mortality and incidence

**Fig. 2:**
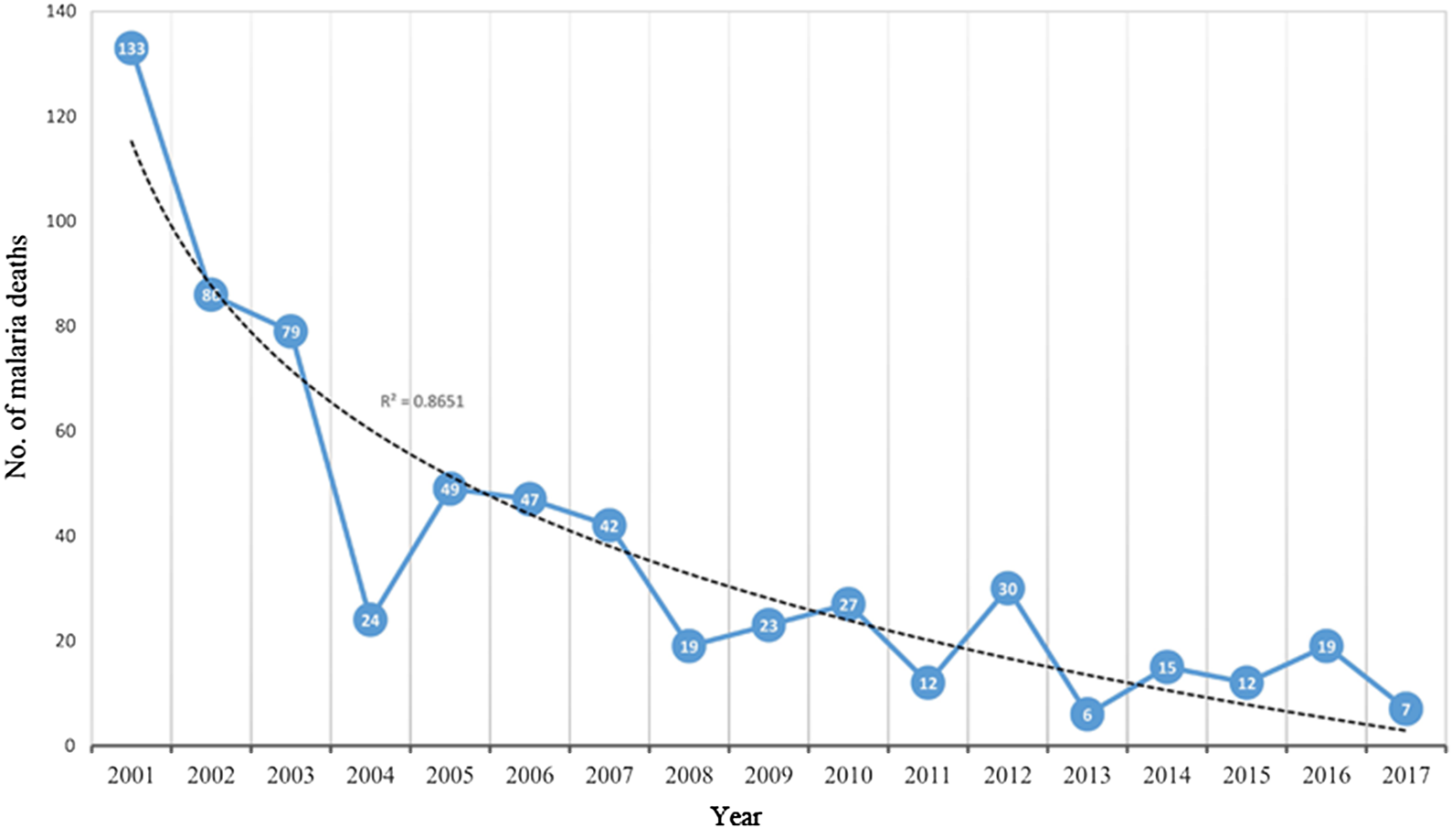
Number of malaria deaths, 2001–2017 (malaria mortality reductions achieved between 2001 and 2017)

**Fig. 3:**
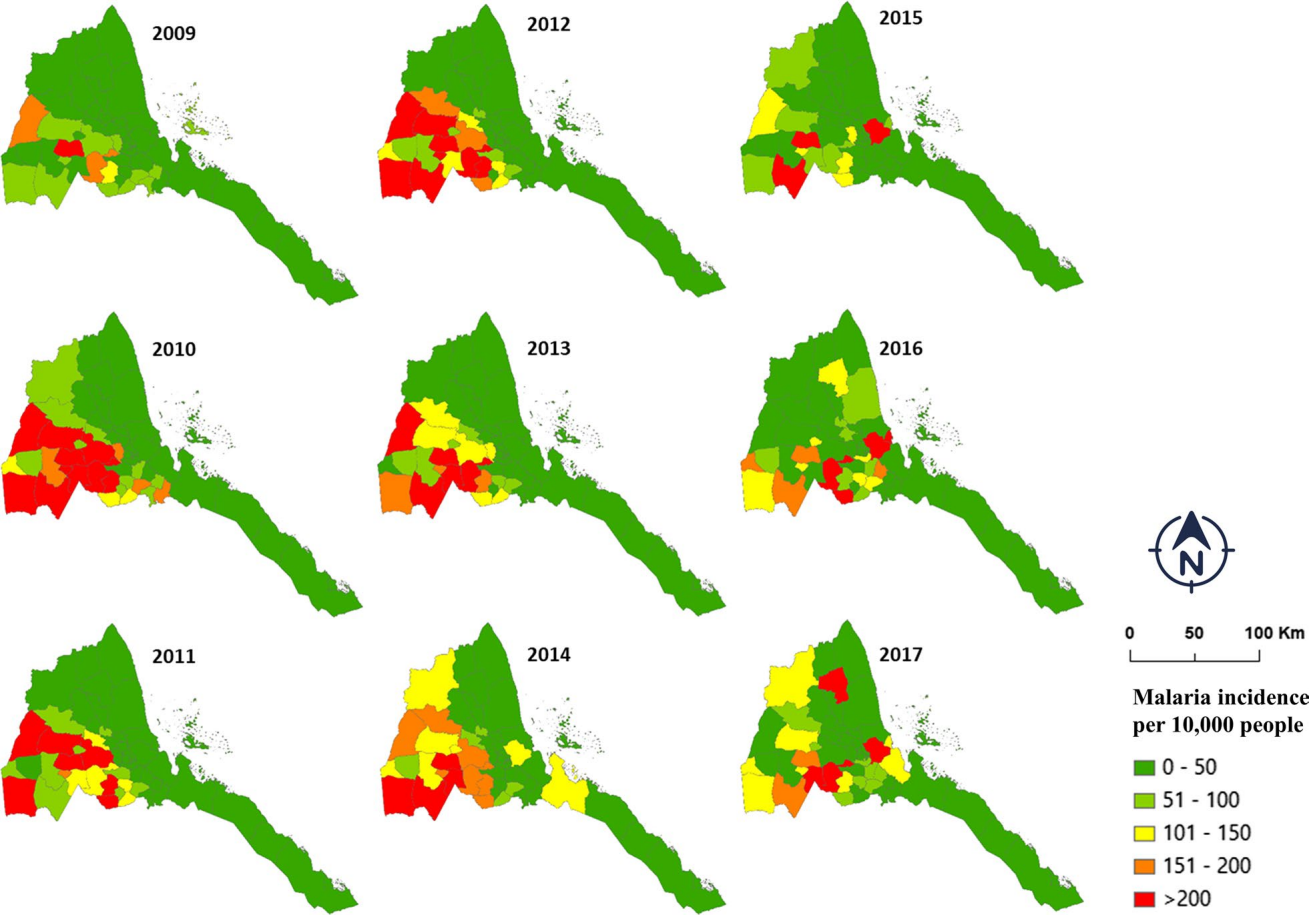
Spatial distribution of malaria incidence in Eritrea, 2009–2017

Considering 2001 as a baseline, malaria mortality declined by over 97% (*R*^2^ = 0.61) by the end of 2017. However, the reported decline was not consistent over the years. Figure 2 shows that while the progress oscillated between malaria gains and losses (reductions and increments), it also remained unstable from year to year with occasional consistencies (Fig. 3). Malaria incidence declined (from 33 to 5 per 1000 population) during the same period by over 86% (*R*^2^ = 0.3). Similarly, this decline coupled by an increase in malaria incidence since 2010 dilutes the overall decline recorded.

### Test of multicollinearity

In real data, there is neither perfect multicollinearity nor orthogonality. The determinant of the correlation matrix lies between zero and one (malaria deaths 0.19 and malaria incidences 0.1882), and there might be some degree of multicollinearity in the exploratory section of the model. The existence of multicollinearity may be considered as the departure from the orthogonality. The stronger the departure from orthogonality (the value of the standardized determinant to unity), the stronger the degree of multicollinearity and *vice versa*.

There are several measures which are used to detect multicollinearity in the literature, for example, the determinant method, R-square method, sum of reciprocal eigenvalues, Farrar-Glauber Chi-square method and Red indicator [22, 23]. All these tests the null hypothesis of orthogonality (no correlation) among explanatory variables. We ran the Farrar-Glauber Chi-square test on the data, 648 classes (months of six zones over the period 2009–2017). Results showed that, on average, one class contributes only 1.65 (square of standard normal variate) for a single class.

### Model validation

We computed the mean square error (MSE) which shows the goodness-of-fit in a model. The estimated MSE determines the statistical significance of the factors or predictors under study. The MSE measures the quality of an estimator being used. The MSE is always positive because of squaring effects of deviation from model estimates. However, this measure does not account for information that could produce a more accurate estimate. Additionally, the MSE varies when there is a high variation in the error magnitude.

For validation purposes, we fitted the model using 80% of the dataset and validated on the remaining 20% by computing ∑(Yi–Yi^)^2^/n. Table 1 shows all MSEs for various models of mortality and incidence. A good model will be one that has a smaller MSE. The model using LLIN as covariate only is the best predicting model for malaria incidence (MSE = 1.17, see Table 1); on the other hand, the model with covariate temperature only is the best predictor for deaths due to malaria.

**Table 1:** Malaria risk factors in Eritrea

Model	Malaria incidence			Mortality		
	MSE	Covariate	*F*-statistic (*df*)	MSE	Covariate	*F*-statistic (*df*)
All covariates model	1.2256	LLIN	6.0664 (1,102)***	1.3972	LLIN	3.8997 (1, 102)
		Population	3.7908 (1, 102)***		Population	1.9997 (1, 102)
		IRS	27.6361 (1, 102)***		IRS	0.0597 (1, 102)
		Temperature	1.2455 (1, 102)***		Temperature	3.8494 (1, 102)*
		Rainfall	8.7734 (1, 102)***		Rainfall	8.8578 (1, 102)**
LLIN and IRS	1.2707	LLIN	2.9653 (1, 105)***	1.3065	LLIN	3.0643 (1, 105)
		IRS	7.5076 (1, 105)***		IRS	7.2561 (1, 105)**
Temperature and rainfall	1.3754	Temperature	4.0844 (1, 105)***	1.2548	Temperature	4.1961 (1, 105)*
		Rainfall	9.4064 (1, 105)***		Rainfall	9.7945 (1, 105)**
Only LLIN	1.1656	LLIN	4.8180 (1, 106)***	1.3947	LLIN	8.4972 (1, 106)*
Only IRS	1.6138	IRS	9.7032 (1, 106)***	1.3237	IRS	9.9540 (1, 106)**
Only temperature	1.2121	Temperature	1.4810 (1, 106)***	1.2307	Temperature	1.6129 (1, 106)
Only rainfall	1.2917	Rainfall	6.2400 (1, 106)***	1.2613	Rainfall	6.2488 (1, 106)*

*P < 0.05, **P < 0.001, ***P < 0.0001

*Note*: *P*-value= Probability (*F*_(1,error df)_ ≥ *F*_calculated)

*Abbreviations*: MSE, mean square error; LLIN, long-lasting insecticidal nets; IRS, indoor residual spraying; df, degrees of freedom

The measure MSE just provides an estimate for a percentage of validation. It might be different for a different validation percentage. Moreover, it always selects a random sample for the training set and the remaining are used for the validation (test) set. Results of MSE might be different if the same percentage of training and test set is used on various runs. This is because of the selection of random samples (splitting data into train set and test set).

### Distribution and use of malaria diagnostic tools

A downward trend of malaria deaths was reported in Eritrea from 2001 to 2017 (Fig. 3). During the same period, a steady increase of suspected fever cases was diagnosed using microscopy and RDTs (Additional file 4: Figure S1). Meanwhile, the observed significant increase in the number of tested suspected malaria cases using microscopy and RDT denotes an equivalent reduction in cases treated based on clinical diagnosis alone.

### Vector control and reported burden of malaria at national and regional level

Since 2001, IRS, LLIN, and larviciding have been the primary intervention tools for targeting vector control. To strengthen case management, Artemisinin Combination Therapy (ACT) and RDT were also introduced at the community level in 2007 [4]. The introduction and subsequent use of RDTs and ACTs have been increasing while the implementation of IRS and LLINs had not declined, as shown in Additional file 4: Figure S2. Meanwhile, the malaria mortality rate significantly reduced during the same period (Additional file 4: Figure S3).

The distribution of LLINs generally declined between 2001 and 2014 (*R*^2^ = 0.16) and increased coverage afterward, while the number of people protected by IRS slightly increased (*R*^2^ = 0.27). LLIN coverage was associated with the reduction of malaria cases whereas an increase in IRS was associated with increased malaria cases (Table 1). However, only LLIN was significantly (95% CI: − 1.07–0.06) associated with predicted deaths. A higher temperature was associated with reduced malaria cases whereas increased rainfall was positively associated with increased number of malaria cases. A low temperature in the rainy season was conducive for malaria spread. Changes in both temperature and rainfall were highly significant while predicting the number of malaria cases and deaths. The corrected AIC (AICc) of a log-likelihood model penalized for several covariates.

**Fig. 4:**
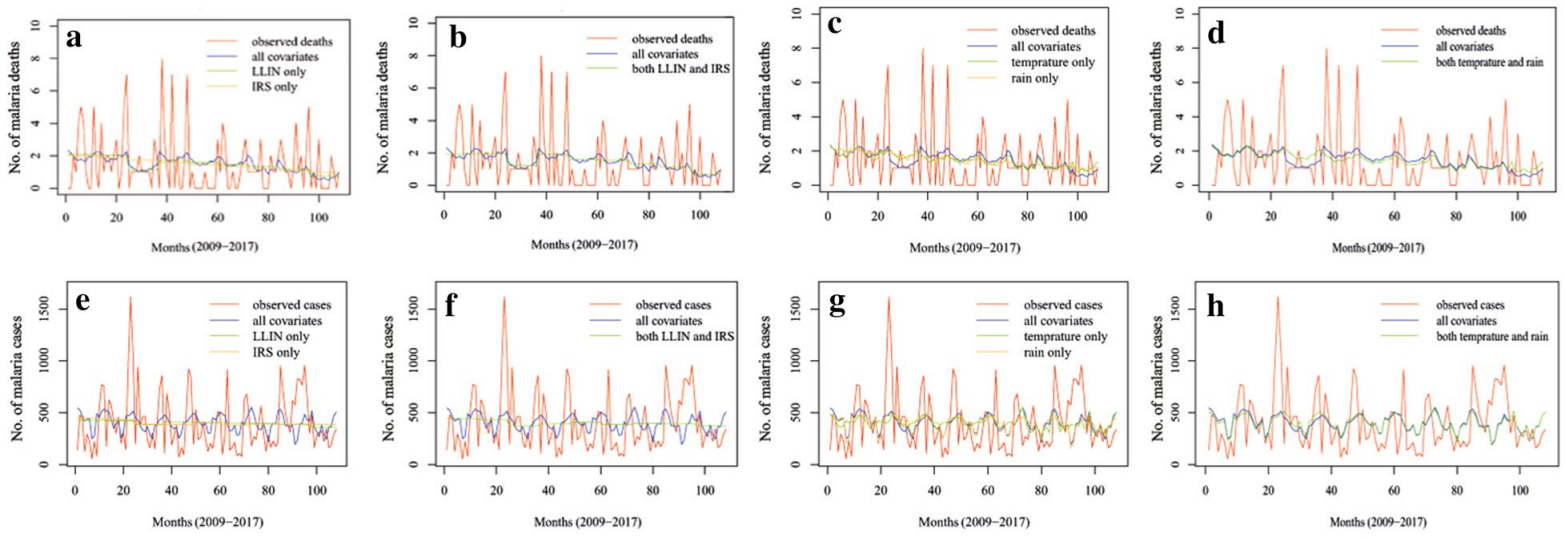
**a** LLIN and IRS as individual predictors of malaria deaths. **b** LLIN and IRS as compound predictors of malaria deaths. **c** Temperature and rainfall as compound predictors of malaria deaths. **d** Temperature and rainfall as individual predictors of malaria deaths. **e** LLIN and IRS as individual predictors of malaria cases. **f** LLIN and IRS as compound predictors of malaria cases. **g** Temperature and rainfall as compound predictors of malaria cases. **h** Temperature and rainfall as individual predictors of malaria cases

Based on Fig. 4 (a–h), the model using all covariates is the best fit (lowest AICc = 359.33). The vector of predicted number of cases by this model has the highest probability of appearing as a predicted vector from all given models. LLIN and IRS (Fig. 4e) is the second likely close line to the actual cases line. This suggests that it is the correlation of a covariate or set of covariates, which makes a covariate efficient predictor.

The introduction of ACTs in 2007 may explain the spatiotemporal distribution (Additional file 4: Figure S4) and change detected in the subsequent years (Additional file 4: Figure S5). Additional file 4: Figure S6 shows the evaluation of the effectiveness of the population-wide health intervention.

The observed change(s) detects whether cases increased or decreased (probability of an event occurring e.g. death) is significantly high (low i.e. different) at that point in time (Additional file 3: Table S2). The Buishand U-test and time series homogeneity tests allow a change point to be detected (Additional file 3: Table S2). The Buishand U-test is more sensitive than the Buishand range test and detects whether cases increased or decreased in a certain time period.

## Discussion

Through its control efforts, Eritrea has made tremendous progress in the fight against malaria in the past decade. The 2009–2017 effective malaria control programme adopted an intervention approach that strengthened vector control, and effective case management [13, 24]. These strategies, coupled with a good reception from the community, resulted in a significant drop in malaria cases [13].

The foregoing observation is not surprising given the 2011 survey finding of no effect on malaria incidence even after the introduction of IRS in Gash-Barka despite the area having high net coverage and low malaria incidence [16, 19].

The change point for the mortality rate in 2007 could be associated with the introduction of RDTs at the national scale that might be related to diagnosis and treatment at the community level. Although this huge drop was attained earlier, the country did not move to eliminate malaria due to various reasons, ranging from failure to sustain such progress, operational challenges in implementing interventions and the lack of a deliberate plan to move from control towards elimination.

In the latter years, the increased IRS and LLINs coverage compared to the past did not result in a continued reduction in cases or even deaths. This is because vector control was applied to the more seasonal or endemic regions where malaria transmission remains in pockets of persistent areas. Thus, although there is no significant reduction in IRS or LLIN, efficiency has improved by targeting high-risk populations and ensuring high intervention coverage to yield the result.

As morbidity and mortality further reduced, cycles of surge periods became shorter (Figs. 2, 3). This is a sign of making progress toward zero cases, and such frequent surges might be due to a loss of immunity as the naive population increases. These gains require more strategic approaches strengthening surveillance, monitoring, and a functioning rapid reporting system.

The benefits from optimal RDTs at limited capacity [24], and the rational use of anti-malarial drugs [25] marked the move away from clinical treatment [26]. Missing the infected individuals driving residual transmission (individuals with clinical immunity) or potential sequestration [27, 28] of *P. vivax*, potentially explains the unstable malaria incidence and resultant epidemics or outbreaks in the latter years. A small fraction of clinical diagnosis implies that it could increase drug resistance due to misdiagnosis and overtreatment [25, 29]. It will also be challenging and take longer to reach a stable suppression of malaria transmission and transmission tail. A transmission tail implies that it requires more investment and resilience because any drop-off would cause disease resurgence due to the population’s waning immunity from lack of exposure. This is not surprising because malaria becomes more elusive as transmission reduces to very low levels usually deemed suitable for elimination, while the efficiency of currently available tools becomes compromised.

Eritrea can drastically reduce the number of malaria cases or even move towards elimination. The country’s move to conducting regular therapeutic efficacy studies [26], insecticide resistance monitoring [30], case management, improved diagnosis [31], mobilization of trained staff [32] and financial resources [11], regularly updating risk maps [33–38], monitoring and evaluation [18, 30, 39–43], can help to sustain the gains and achieve elimination.

Resource allocation tools and methods must be strategically applied in order to balance control in endemic zones such as Gash-Barka and prevent reintroduction in areas with low to zero cases. Likewise, behavioral change communication remains essential for the population to stay alert and ensure continuity in the utilization of preventive interventions such as LLINs until an informed decision is made by the government to withdraw or halt malaria prevention services from a certain area. Given environmental management, LLINs and IRS, complimented with larviciding in certain areas, remain the only vector control interventions being implemented in the country. With current plans focusing on elimination through testing, treating, documentation, reporting and tracking of every fever/malaria case (WHO’s Test, Treat and Track approach), Eritrea must introduce mobile phone technology for real-time reporting of malaria cases from all health facilities [44].

We used routinely collected malaria cases data for this study. Many cases were not confirmed by RDT and microscopy but were based on clinical signs and symptoms. Both RDT and microscopy have limited sensitivity and specificity and are particularly likely to misclassify individuals with low levels of parasitemia. HRP2-detecting RDTs report false negative results on *P. falciparum* species due to deletion of the *Pfhrp2* gene as shown by Pati et al. [45] in 2015. Finally, the census populations used were derived from estimates because there has not been any census in Eritrea since 1994. Despite all these challenges, this study documented the progress and identified challenges to control and eliminate malaria in Eritrea with longer time series data. Any variation generated by the general discussion of malaria without speciation (*P. vivax vs P. falciparum*) is covered by evidence [46] that diagnostic tests (microscopy and RDTs) were able capture both invariably.

## Conclusions

The progress achieved by Eritrea towards malaria elimination should be further maintained and improved. Efforts should be sustained to keep effective malaria control and eventual elimination of the disease in the country. Distribution, coverage and utilization of malaria control and elimination tools should be optimized and sustained to safeguard these gains. Consistent annual performance evaluation of malaria indicators would ensure a continuous learning process from gains/threats of epidemics and resurgence in regions already earmarked for elimination. As elimination of malaria becomes more imminent, a stronger malaria surveillance system will also need to be well equipped to tackle potential malaria case importation. Hence, prior operationalization of existing interventions and efforts will go a long way towards elimination. It will require sustained support and commitment from the National Malaria Control Programme, as well as political leaders coupled with adequate local resource mobilization.

## Supplementary information

**Additional file 1: Table S1.** Milestones of malaria control programme in Eritrea.

**Additional file 2: Text S1.** Additional methods and climatic parameters by geographical region.

**Additional file 3: Table S2.** Change detection tests to confirm time points with identifiable significant changes.

**Additional file 4: Figure S1.** Trends in malaria cases and method of diagnosis in Eritrea, 2001 to 2017 (* No data available for the number tested by both RDT and microscopy in 2016 and 2017). **Figure S2.** Trends in type and quantity of interventions in relation to mortality, 2001 to 2017. **Figure S3.** Malaria incidence, admissions and mortality rates, 2001 to 2017. **Figure S4.** Spatial distribution of malaria prevalence in Eritrea, 2009 to 2017. **Figure S5.** Eritrea malaria incidence, 2001–2017 after introducing ACTs in 2003 (as change point). Interrupted time series with a level change regression model, and a (line) predicted trend using the unadjusted regression model. **Figure S6.** Malaria incidence in Eritrea, 2001–2017, step *vs* step change-only models. No significant difference was detected using the F-test, which accounts for the over-dispersion.

## Data Availability

Data supporting the conclusions of this manuscript are provided within the article and its additional files and original datasets are available from the corresponding author upon request.

## Abbreviations

ACT: artemisinin combination therapy
ALMA: African Leaders Malaria Alliance Award
CFSR: climate forecast system reanalysis
CHAs: community health assistants
CHIRPS: Climate Hazards Group Infrared Rainfall with Stations
HMIS: Health Management Information System
HQ: headquarters
IRS: indoor residual spraying
LLIN: long-lasting insecticidal nets
MDGs: Millennium Development Goals
NCEP: National Centers for Environmental Prediction
NMCP: National Malaria Control Programme
RDT: rapid diagnosis test
TPR: test positivity rate
USGS: United States Geological Survey
WHO: World Health Organization
