# Supplementary material for: Retrospective data analyses of social and environmental determinants of malaria control for elimination prospects in Eritrea
Source: Parasit Vectors. 2020 Mar 12;13:126. doi: 10.1186/s13071-020-3974-x (PMC7068948; doi:10.1186/s13071-020-3974-x)
Supplement: Supplementary file 1 — Additional file 1: Table S1. Milestones of malaria control programme in Eritrea. [file 13071_2020_3974_MOESM1_ESM.docx]

**Additional file 1: Table S1. Milestones of malaria control program in Eritrea**

| **YEAR** | **MILESTONES** |
| --- | --- |
| 1995 - 1997 | - 1995 Establishment of the National Malaria Control Program (NMCP) - 1997 First 3-year National Malaria Strategy drafted |
| 1998 | - 1998 National malaria epidemic occurred - 1998 First national RBM meeting conducted which came out with a declaration of reducing morbidity & mortality by 80% (‘Mendefera Declaration’) |
| 2001 - 2003 | - 2000 Second National Strategic Plan launched (2000-2004) - 2001 Launch of the HAMSET Control Project for HIV/AIDS, Malaria TB and Reproductive Health - First Malaria Prevalence Survey conducted - 2002 First Case Management guideline disseminated - 2002 Shift in treatment of uncomplicated malaria cases, from chloroquine to chloroquine+sulfadoxine-pyrimethamine combination. - 2003 Restructuring of the Ministry of Health and the creation of the Communicable Diseases Control Division (CDC) within the Ministry of Health which included Malaria, IDSR and NCDs - 2003 A Malaria Policy launched - 2003 Global Fund Round 2 grant approved |
| 2004 - 2006 | - 2004 Malaria Indicator and Health Facility surveys conducted - 2004 Three Insectaries were established for malaria vector monitoring and surveillance, funded by World Bank - 2005 Third National Strategic Plan launched (2005-2009) - 2006 Global Fund Round 6 grant approved - 2006 A combination of Sulfadoxine-pyrimethamine and Chloroquine discontinued as treatment option for uncomplicated malaria cases - 2006 RDT fully scaled up from the pilot phase |
| 2007 - 2009 | - 2007 A combination of artesunate+amodiaquine introduced as treatment of choice for uncomplicated malaria cases. - 2007 RDTs introduced at community level (CHAs) - 2008 Malaria Case Management guideline revised - 2008 MIS and health facility surveys - 2009 Fourth National Malaria Strategic Plan launched (2010-2014) |
| 2010 - 2013 | - 2010 Global Fund Round 9 grant approved - 2012 Shift from DDT to carbamates (Bendiocarb 80%WP) and pyrethroids for Indoor Residual Spraying - 2012 Malaria Program Review conducted - 2013 Effectiveness of Malaria Communication Strategies studied - 2013 Study on socio-economic determinants of early treatment seeking and use of bed nets |
| 2014 - 2015 | - Concept Note for Global Fund Malaria Grant 2015-2017 Submitted and Approved - National Malaria Treatment Guideline revised (2015) - WHO Malaria Case Management Training Manual adopted (2016) - Insecticide Resistance Management Plan drafted (2016) - IVM guideline revised (2015) - Larval Source Management Guideline drafted (2015) - HRP-II antigen deletion from P. falciparum detected and new rapid tests based on pLDH antigen identified. - 2015 Extrapyramidal reactions detected in malaria patients attributable to administration with artesunate+amodiaquine tablets. |
| 2016 - 2018 | - Jan 2016 – Eritrea received ALMA award of excellence in malaria control - Artesunate injection introduced for treatment of severe malaria - Nov. 2016 HRP2 based RDTs replaced with Pf-pLDH/PanpLDH RDTs - Jul-Aug 2018 Pirimiphos-methyl 300CS applied for Indoor Residual Spraying for the first time. - 2018 Risk Management Plan launched in response to adverse drug reactions (Extrapyramidal reactions) in patients' post-treatment with artesunate+amodiaquine - 2018 Malaria indicator survey and health facility survey conducted - 2018 Vector behaviour study conducted - 2018 Molecular markers of drug resistance – under study |
