# Supplementary material for: Retrospective data analyses of social and environmental determinants of malaria control for elimination prospects in Eritrea
Source: Parasit Vectors. 2020 Mar 12;13:126. doi: 10.1186/s13071-020-3974-x (PMC7068948; doi:10.1186/s13071-020-3974-x)
Supplement: Supplementary file 2 — Additional file 2: Text S1. Additional methods and climatic parameters by geographical region. [file 13071_2020_3974_MOESM2_ESM.docx]

## Additional file 2: Text S1. Additional methods and climatic parameters by geographic regions

## METHODOLOGY

## Overview of the laboratory system

The primary level of the health system comprises of community-based health services; health Stations which deliver facility-based primary health care services and oversee community-based activities and community hospitals that serve as referral units for the health stations. The secondary level of services is made up of the Zonal referral hospital laboratories and second contact hospital laboratories, while the tertiary level of service is comprised of the national referral hospital laboratories and National Health Laboratory which also serve as national centres of excellence for specialized testing, training/ education, research and continuing education.

Laboratory personnel are mainly of three categories: Clinical laboratory scientists (medical laboratory technologists) who are holders of Bachelor of Science degree, medical laboratory technologists who are holders of diploma and laboratory technicians who are holders of a one year college certificates. RDT providers are mainly community health agents and Associate nurses.

**Eritrea National laboratory referral system**

### Rapid Diagnostic Tests

In accordance to the recommendations, Eritrea started to use RDTs for malaria diagnosis in 2007 to bridge the shortage of microscopy services at health facilities. These have been used with very good results and minimized the number of cases that were treated clinically at all levels and improved rational use of anti-malarial drugs. The RDTs were later on (in 2008) rolled out to community health agents in all the zones. In the initial period, the type of RDTs that was deployed was of a *P. falciparum*-only type, which was later on replaced with tests detecting both *P. falciparum* and *P. vivax* (*Pf*hrp2/pLDH). As a result, a shift has been made to the best option available as of November 2016, which is Pf/Pan (pLDH based) RDTs that can detect falciparum and non-falciparum infections.

RDTs are mainly performed at Health stations by nurses/associate nurses and Community level by community health agents. Each community health agent is linked to a health station or health centre. RDTs are also performed in the laboratories whenever microscopy is temporarily unavailable. RDTs are performed mostly from finger prick but also EDTA venous blood. Results are reported as Plasmodium falciparum positive or Plasmodium vivax positive or Malaria Negative or as per manufacturer’s instructions.

###

### Microscopy

Microscopy is performed in all health centre laboratories, second contact hospital laboratories and Zonal hospital laboratories. Microscopy is issued for initial diagnosis, monitoring of treatment and drug efficacy studies. It is performed from both thick and thin smears. All malaria microscopy results are recorded in the malaria microscopy registers.

**Gash Barka Zone:** Has a July-September rainfall season and a small rain season in April/May. The drivers of transmission are the many brick making sites, the big rivers, the border with Ethiopia and Sudan, many inaccessible areas, traditional mining sites, lots of dams and irrigation activities (gash Barka is the bread basket of the nation). This is coupled with movement of non-immune people to these areas for economic activities which may worsen the malaria situation.

**Debub Zone:** Has July-September rainy season as well as small rain in April/May. Divers of transmission are like those in Gash Barka. It also shares border with Ethiopia but limited movement of people across the borders limits cross-border transmission of malaria here. Debub has the highest population density in the country. Some areas are difficult to access. Debub is a large Zone like Gash Barka- the result is management challenges. There are also traditional mining sites in Debub.

**Anseba Zone:** Has a July-September rainy season as a well as the April/May small rain. This zone has imported malaria cases from Gash Barka. There are lots of dams and rivers. They have good surveillance and know that about 60% of malaria cases in the Zone are imported from Gash Barka.

**Northern Red Sea Zone**: Has a December/January raining season with no April/May small rain. The land mass is very big but malarious areas are limited because the coastal area is desert and very hot. Has rivers and two raining seasons in some areas.

**Southern Red Sea Zone:** Has December/January rainy season and no April/May small rain. Cases are found along the rivers; there is perhaps no local transmission and cases imported from the highlands. Have larvae eating fish here.

**Maekel Zone:** Has July-September rainy season as well as the small rain of April/May. The malaria cases in tis zone are mainly imported but there are few villages with local transmission.
