# Supplementary material for: Retrospective data analyses of social and environmental determinants of malaria control for elimination prospects in Eritrea
Source: Parasit Vectors. 2020 Mar 12;13:126. doi: 10.1186/s13071-020-3974-x (PMC7068948; doi:10.1186/s13071-020-3974-x)
Supplement: Supplementary file 3 — Additional file 3: Table S2. Change detection tests to confirm time points with identifiable significant changes. [file 13071_2020_3974_MOESM3_ESM.docx]

**Additional file 3: Table S2. Change detection tests to confirm time points with identifiable significant changes**

| **Variable** | **Change-Point Detected at time t** | **Year of change point** | **Buishand range test (p-value)** | **Buishand U test (p-values)** | **Any special event** |
| --- | --- | --- | --- | --- | --- |
| **Malaria admissions** | 3 | 2003 | 0.258 | 0.021* | *ACTs introduced in 2007 |
| **Malaria admission rate** | 3 | 2003 | 0.152 | 0.009* | *ACTs introduced in 2007 |
| **Total confirmed & clinical cases** | 3 | 2003 | 0.112 | 0.041* | *ACTs introduced in 2007 |
| **Malaria deaths** | 3 | 2003 | 0.088 | 0.001* | *ACTs introduced in 2007 |
| **Malaria incidence rate** | 3 | 2003 | 0.1039 | 0.027* | *ACTs introduced in 2007 |
| **Malaria mortality rate** | 7 | 2007 | 0.0751 | 0.001* | *RDTs scale up 2007 |
| **Confirmed malaria cases** | 9 | 2009 | 0.005** | 0.0016* |  |
| **Total Tested (Mic + RDTs)** | 9 | 2009 | 0.027** | <0.0001* |  |
| **Test positivity rate (TPR)** | 9 | 2009 | 0.191 | 0.606 |  |
| **Positive** | 9 | 2009 | 0.003*** | 0.002* |  |
| **#of people protected by IRS** | 10 | 2010 | 0.470 | 0.094 |  |
| **# of ITNs distributed** | 10 | 2010 | 0.6565 | 0.7133 |  |
| **ABER** | 10 | 2010 | 0.1798 | 0.011* |  |

* Result is significant at 95% CI.
